# Sleep Disturbance as a Catalyst in the Cyclical Link Between Depressive Symptoms and Disability in Instrumental Activities of Daily Living in Older Chinese Adults: Longitudinal Cohort Study

**DOI:** 10.2196/76643

**Published:** 2025-11-06

**Authors:** Hao Wu, Lu Wang, Xin Liu, Yuyue Yang, Wei Gao

**Affiliations:** 1 Department of Epidemiology and Health Statistics, School of Public Health, Jiangxi Medical College, Nanchang University, No. 1299 Xuefu Avenue, Nanchang, 330031, China, 86 0791-86362275; 2 Jiangxi Provincial Key Laboratory of Disease Prevention and Public Health, Nanchang University, Nanchang, China

**Keywords:** sleep disturbance, depression, disability in IADLs, cross-lagged panel model, longitudinal mediation, older adults

## Abstract

**Background:**

Depressive symptoms, sleep disturbances, and functional disability are interrelated. However, the bidirectional pathways between depression, sleep disturbances, and disability in instrumental activities of daily living (IADLs) remain underexplored in China.

**Objective:**

We aimed to examine the bidirectional longitudinal relationships between depression and disability in IADLs among older Chinese adults, with a focus on elucidating the mediating role of sleep disturbances in this dynamic interplay.

**Methods:**

The study encompassed 2677 older adults who provided complete data at T1 (2015), T2 (2018), and T3 (2020) for the China Health and Retirement Longitudinal Study (CHARLS). Depressive symptoms were assessed using the 10-item Center for Epidemiological Studies Depression (CESD-10) scale, and a 6-item scale was used to measure disability in IADLs. Sleep disturbances were self-reported. Temporal associations between depressive symptoms and disability in IADLs as well as the longitudinal mediating effect of sleep disturbances were examined using a cross-lagged panel model.

**Results:**

Prior depression significantly predicted subsequent disability in IADLs at T2 (*β*=0.070, *P*<.001) and T3 (*β*=0.074, *P*<.001), and prior disability in IADL predicted subsequent depression at T2 (*β*=0.094, *P*<.001) and T3 (*β*=0.100, *P*<.001). Additionally, the indirect effect of prior disability in IADLs on subsequent depression via sleep disturbances was statistically significant (*β*=0.062, SE=0.010, *P*<.001), with the mediation effect accounting for 50.41% of the total effect. In contrast, after accounting for this mediation, the direct effect of prior depression on subsequent disability in IADLs was not significant (*β*=0.009, SE=0.018, *P*=.61). Consequently, the impact of depression on disability in IADLs was fully mediated through sleep disturbances in this cohort of older Chinese adults.

**Conclusions:**

Depressive symptoms and disability in IADLs are bidirectionally linked, and sleep disturbances play a longitudinal mediating role in the bidirectional relationship among older Chinese adults. The potential longitudinal bidirectionality highlights the importance of sleep health for interventions on depression and functional disability in older adults.

## Introduction

According to the World Health Organization, the number of people aged 60 years and older outnumbered children younger than 5 years in 2020. Between 2015 and 2050, the proportion of the world’s population older than 60 years will nearly double from 12% to 22% [1]. China’s accelerated demographic transition since achieving aging society status 2 decades ago coincides with growing global health challenges, including cognitive impairment and physical frailty.

Aging, as an inescapable natural process, progressively influences our functional capacities, mental health, a growing risk of disease, and social interactions. Functional disability affects more than 1 billion people worldwide according to the 74th World Health Assembly, and this number is increasing as the aging population increases [2]. Activities of daily living (ADLs) and instrumental activities of daily living (IADLs) are key measures, with ADLs covering basic self-care and IADLs involving more complex tasks necessary for independent living and social participation [3,4]. Although there is some debate as to whether ADLs or IADLs reflect separate constructs, most conceptualizations assume a hierarchical relation between the 2 constructs, with disability in IADLs preceding disability in ADLs [5]. Therefore, impairments of IADLs may indicate an early decline in physical function [6]. The mental health of older adults, particularly depression, demands urgent attention. With approximately 4.7% of the global population experiencing a depressive episode annually [7] and rates in Chinese people older than 45 years reaching 26.67% for men and 38.37% for women [8], the rising prevalence of depressive symptoms poses a significant public health challenge. Late-life depression, linked to physical illness, functional impairment, and mortality [9], underscores the need for identifying modifiable risk factors. Sleep, a potentially modifiable lifestyle behavior, plays a very important role in health and well-being. Nearly one-half of the world’s population experience sleep disturbances and an estimated one-third of adults have experienced insomnia symptoms [10]. Further, older adults may under-report the severity of sleep disturbances compared with objective assessments by polysomnography [11]. Both sleep disturbances and depression are associated with a greater risk of adverse health-related outcomes, including poor health status [12], disability [13], and poorer physical functioning in older populations [14].

Currently, multiple cross-sectional and longitudinal studies have confirmed the pairwise bidirectional associations between sleep, depressive symptoms, and functional disability in older adults globally [15-17]. A study with older adults aged 60 years and older in 3 provinces in China showed that the effect of limitations in IADLs on depressive symptoms in older adults was greater than that of limitations in ADLs [18]. Additionally, a bidirectional association between sleep disturbances and depression was found in older adults [19,20], in which sleep disturbances might exacerbate depression, and older adults with depression often experience poor sleep quality [21,22]. Some studies found that short [14] or long [23] sleep duration was associated with disability in IADLs, whereas others showed that both sleep durations were related to disability in IADLs in older people [24]. Older adults with more limitations in ADLs and IADLs had a higher risk of experiencing declines in sleep quality and the transition from meeting to not meeting the recommended sleep duration over time [25].

Previous studies have indicated that depression can impair IADLs by reducing motivation and exacerbating fatigue, while declines in IADLs may in turn increase the risk of depression [15,16]. Therefore, we hypothesized a bidirectional relationship between depression and disability in IADLs. Furthermore, extensive studies have firmly established significant bidirectional relationships between sleep and depression as well as between sleep and functional disability [20-23,25]. Given that sleep disturbances are particularly prevalent among older adults and could act as both a consequence of depression and a precursor to functional disability, we further proposed that sleep plays a mediating role in the relationship between depression and functional disability. Based on substantial literature, we also suggested that gender differences might lead to distinct stress coping mechanisms and health behavior patterns [26], regional disparities reflect differences in health care accessibility and lifestyle [27], and empty nest status—as an important psychosocial factor—might influence physical and mental health outcomes among older adults [28]. Additionally, our research, using a conceptual framework, had found that sleep significantly mediated this interaction, acting as both a consequence and an active contributor [29]. However, despite these conceptual advances, a critical gap remains. Few longitudinal studies have specifically tested the mediating role of sleep in the bidirectional relationship between depression and functional disability among older adults in China. The Chinese context, characterized by its unique sociocultural dynamics and rapidly aging population, may shape these pathways in distinct ways. Furthermore, the changing morbidity patterns in China warrant a specific focus on IADL-related issues [30]. To address this gap, our study aimed to elucidate this complex interplay by using a cross-lagged panel model (CLPM) and testing 3 main hypotheses: (1) A bidirectional relationship exists between depression and disability in IADLs; (2) sleep disturbances longitudinally mediate this relationship; and (3) the strength of these associations and the mediating effect vary by gender, district, and empty nester status.

## Methods

### Data Collection and Study Participants

This study used data from the China Health and Retirement Longitudinal Study (CHARLS), a national longitudinal study of adults aged ≥45 years across 28 provinces in China. Initially conducted in 2011, it included 5 waves of data collection as of publication of this study. The CHARLS was approved by the Biomedical Ethics Review Committee of Peking University [31], and informed consent was obtained from all participants. Detailed information on study sampling, including the inclusion and exclusion criteria, can be found in earlier publications [31]. For this study, data from 2015, 2018, and 2020 were used, focusing on individuals aged ≥60 years without cognitive impairments (score<1.5 SD [32]) or missing data on sleep, depression, or disability in IADLs. Ultimately, a total of 2677 participants were included in this study (Multimedia Appendix 1). Reporting follows the Reporting of Studies Conducted Using Observational Routinely-Collected Health Data (RECORD) guidelines (Checklist 1).

### Measurements

Depressive symptoms were assessed using the 10-item Center for Epidemiologic Studies Depression (CESD-10) scale, with scores ranging from 0 to 30. Participants were defined as having a higher sensitivity for clinically significant depressive symptoms if they scored 12 or higher on the CESD-10 scale [33]. IADLs, as assessed using the Lawton IADL Scale, represent the abilities required for community living that depend on advanced cognitive functioning [6]. The final score was a sum of responses to the 6 items and ranged from 0 to 18 points: the higher the score, the higher the level of disability in IADLs for the individual. Sleep disturbances were assessed using a single-item sleep quality scale and nighttime sleep duration, which were both commonly used in previous studies [34]. The total score for sleep disturbances was calculated as the sum of the scores for the single-item sleep quality scale and nighttime sleep duration, ranging from 0 to 6. It is important to note that this composite measure captured subjective sleep disturbances and was not equivalent to a clinically diagnosed sleep disorder. Although this approach had not been formally validated against established instruments such as the Pittsburgh Sleep Quality Index or objective measures (actigraphy), similar methods were used in prior research using the CHARLS data [34]. Detailed information is in Multimedia Appendix 1.

### Covariates

To minimize the potential confounding variables influencing the relationship between depressive symptoms, sleep disturbances, and disability in IADLs and to keep the model simplicity, limited covariates were adjusted. Covariates were selected based on the established theoretical and empirical associations with depressive symptoms, sleep disturbances, and functional disability in the existing literature on aging populations [6,15-17,22,23,25]. The covariates consisted of sociodemographic characteristics (age, gender, marital status, education, empty nester status, district, and working status), lifestyle behaviors (smoking and drinking status), cognitive function, and the number of noncommunicable diseases. Sociodemographic factors and lifestyle behaviors are fundamental determinants of health outcomes. The number of noncommunicable diseases was included as a proxy for overall physical health burden, a key potential confounder. Cognitive function was adjusted for due to its close linkages with both depression and the ability to perform IADLs [14,24]. Detailed information is in Multimedia Appendix 1.

### Statistical Analysis

Demographic characteristics of the participants were analyzed and presented as frequencies and proportions for categorical variables and means and SDs for continuous variables. To study the causal relationships between depressive symptoms, disability in IADLs, and sleep disturbances over time, a CLPM with maximum likelihood estimation was conducted [35]. The CLPM with observed variables evaluated the association between depressive symptoms and disability in IADLs at all time points and the mediating role of sleep disturbances, with 4 models constructed with varying constraints on parameters. We tested 4 models: Model 1a-2a, an unconstrained CLPM adjusted for covariates; Model 1b-2b, with constrained cross-lagged parameters; Model 1c-2c, with constrained autoregressive parameters; and Model 1d-2d, with both autoregressive and cross-lagged parameters constrained. Model fit was evaluated using the root mean square error of approximation (RMSEA), comparative fit index (CFI), standardized root mean square residual (SRMR), and Tucker-Lewis index (TLI), with specific cutoffs for good fit (Multimedia Appendix 1). A potential conceptual overlap was noted between item 7 of the CESD-10 scale (“My sleep was restless”) and our measure of sleep disturbance. To assess whether this overlap inflated the mediation effects, a sensitivity analysis was performed. All CLPMs were reestimated using a modified CESD-10 score from which item 7 was excluded. The results of this sensitivity analysis are reported in MultimediaAppendices 2  3.

Descriptive statistic analysis was conducted using R software (Version 4.2.2, R Foundation for Statistical Computing). The CLPMs were constructed using Mplus software (Version 8.3, Muthén & Muthén). A significance level of *P*<.05 was used to indicate statistical significance of differences.

### Ethical Considerations

Our study used publicly available anonymized data from the CHARLS and did not require further review. Ethical approval for all the CHARLS waves was granted from the Institutional Review Board at Peking University (IRB00001052-11015). The original ethical approval and participant consent include the use of data. Participants gave informed consent to participate in the study before taking part.

## Results

### Descriptive Statistics

**Figure 1.**
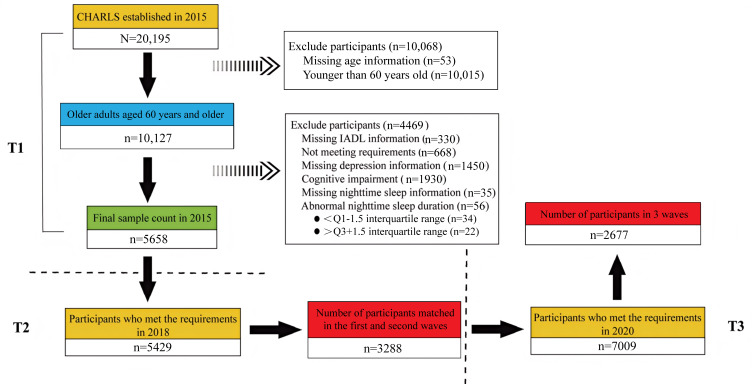
Flowchart of participant selection for the study. CHARLS: China Health and Retirement Longitudinal Study; IADL: instrumental activity of daily living; T1: 2015; T2: 2018; T3: 2020.

A total of 2677 older adults aged 60 years and older participated in this study and completed data collection from 2015 to 2020 (Figure 1). The sample characteristics based on baseline data and the distribution of key variables by period are presented in Table 1. The majority of participants resided in villages (1639/2677, 61.23%) rather than in urban areas (1038/2677, 38.77%), and most were empty nesters (1690/2677, 63.13%). Among the participants, 61.75% (1653/2677) had completed at most a primary education. From T1 to T3, there was a slight decline in the proportion of married older adults, by 5.46%. The number of older adults who smoked was slightly higher than that of nonsmokers (T1: 1395/2677, 52.11% vs 1282/2677, 47.89%; T2: 1424/2677, 53.19% vs 1253/2677, 46.81%; T3: 1417/2677, 52.93% vs 1260/2677, 47.07%), and the majority were nondrinkers (T1: 1590/2677, 59.39%; T2: 1600/2677, 59.77%; T3: 1577/2677, 58.91%). As age increased, the prevalences of depressive symptoms, sleep disturbances, and disability in IADL among older adults also increased.

**Table 1.** Demographic characteristics and changes in depressive symptoms, sleep disturbance, and disability in instrumental activities of daily living (IADLs) from T1 to T3 (N=2677).

Characteristic	2015 (T1)	2018 (T2)	2020 (T3)
Age (years), mean (SD)	65.78 (4.84)	—^a^	—
Gender, n (%)			
Male	1630 (60.89)	—	—
Female	1047 (39.11)	—	—
Empty nester status, n (%)			
Empty nesters	1690 (63.13)	—	—
Not empty nesters	987 (36.87)	—	—
District, n (%)			
Village	1639 (61.23)	—	—
Urban	1038 (38.77)	—	—
Education, n (%)			
Illiteracy	781 (29.17)	—	—
Primary education or less	872 (32.57)	—	—
Junior high school	646 (24.13)	—	—
High school or more	378 (14.13)	—	—
Marital status, n (%)			
Married	2379 (88.87)	2297 (85.81)	2233 (83.41)
Not in marriage	298 (11.13)	380 (14.19)	444 (16.59)
Working status, n (%)			
Government/institutions/firm	147 (5.49)	128 (4.78)	144 (5.38)
Self-employed individual	1207 (45.09)	1179 (44.04)	208 (7.77)
Farmer	161 (6.01)	57 (2.13)	1020 (38.10)
Others	1162 (43.41)	1313 (49.05)	1305 (48.75)
Smoking status, n (%)			
Yes	1395 (52.11)	1424 (53.19)	1417 (52.93)
No	1282 (47.89)	1253 (46.81)	1260 (47.07)
Drinking status, n (%)			
More than once a month	842 (31.45)	838 (31.30)	821 (30.67)
Less than once a month	245 (9.16)	239 (8.93)	279 (10.42)
None of these	1590 (59.39)	1600 (59.77)	1577 (58.91)
Depressive symptoms (score), mean (SD)	8.22 (4.58)	8.85 (4.79)	9.25 (5.25)
Disability in IADLs (score), mean (SD)	0.55 (1.54)	0.68 (1.80)	0.63 (1.75)
Sleep disturbances (score), mean (SD)	1.95 (1.88)	2.13 (1.92)	2.19 (1.93)

a Not applicable.

### The Reciprocal Relationship Between Depressive Symptoms and Disability in IADLs

**Figure 2.**
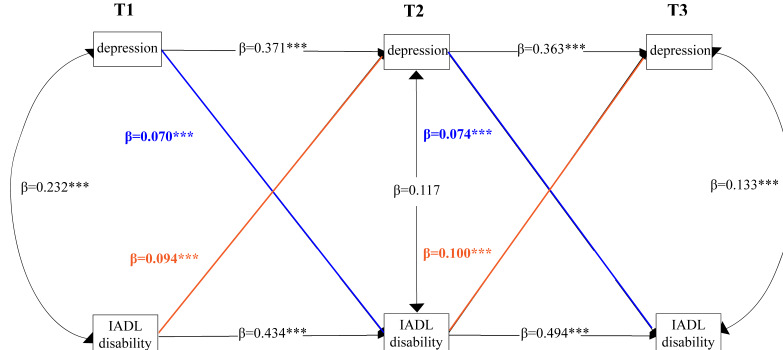
Cross-lagged panel model of the bidirectional relationship between depressive symptoms and disability in instrumental activities of daily living (IADLs). T1: 2015; T2: 2018; T3: 2020. ***P<.001*.*

Figure 2 depicts the CLPM of the bidirectional relationship between depressive symptoms and disability in IADLs (Model 1d). The fit indices for all 4 models are summarized in Multimedia Appendix 4. After controlling for covariates, Model 1d still had a good fit to the data (RMSEA=0.048, SRMR=0.029, CFI=0.903, TLI=0.900). As expected, depressive symptoms at each time point were positively related to depressive symptoms over time, as was disability in IADLs. The cross-lagged effects of prior depressive symptoms on subsequent disability in IADLs at T2 (*β*=0.070, *P*<.001) and T3 (*β*=0.074, *P*<.001) as well as disability in IADLs on subsequent depressive symptoms at T2 (*β*=0.094, *P*<.001) and T3 (*β*=0.100, *P*<.001) were significant (Table 2). Greater prior depressive symptoms predicted greater subsequent disability in IADLs, and greater prior disability in IADLs predicted greater subsequent depressive symptoms. Additionally, the effect of disability in IADLs on depression was greater than the impact of depression on disability in IADLs.

**Table 2.** Statistical results of the bidirectional relationship between depressive symptoms and disability in instrumental activities of daily living (IADLs).

Variable	Autoregression estimates						Cross-lagged estimates					
	Depressive symptoms→disability in IADLs			Disability in IADLs→depressive symptoms			Depressive symptoms→disability in IADLs			Disability in IADLs→depressive symptoms		
	β (95% CI)		*P* value	β (95% CI)		*P* value	β (95%CI)		*P* value	β (95% CI)		*P* value
Model 1a^a^												
2015→2018	0.351 (0.317-0.384)		<.001	0.474 (0.444-0.504)		<.001	0.061 (0.028-0.095)		<.001	0.096 (0.060-0.132)		<.001
2018→2020	0.381 (0.349-0.414)		<.001	0.470 (0.440-0.500)		<.001	0.079 (0.045-0.113)		<.001	0.101 (0.066-0.136)		<.001
Model 1b^b^												
2015→2018	0.351 (0.318-0.384)		<.001	0.472 (0.443-0.502)		<.001	0.068 (0.045-0.091)		<.001	0.095 (0.071-0.119)		<.001
2018→2020	0.380 (0.348-0.412)		<.001	0.472 (0.442-0.501)		<.001	0.073 (0.048-0.098)		<.001	0.102 (0.076-0.127)		<.001
Model 1c^c^												
2015→2018	0.372 (0.347-0.397)		<.001	0.433 (0.410-0.456)		<.001	0.076 (0.043-0.110)		<.001	0.084 (0.049-0.119)		<.001
2018→2020	0.363 (0.335-0.390)		<.001	0.494 (0.469-0.520)		<.001	0.068 (0.035-0.101)		<.001	0.109 (0.075-0.143)		<.001
Model 1d^d^												
2015→2018	0.371 (0.346-0.396)		<.001	0.434 (0.411-0.457)		<.001	0.070 (0.047-0.093)		<.001	0.094 (0.070-0.119)		<.001
2018→2020	0.363 (0.336-0.391)		<.001	0.494 (0.468-0.519)		<.001	0.074 (0.049-0.098)		<.001	0.100 (0.075-0.126)		<.001

a Model 1a: unconstrained model.

b Model 1b: constrained cross-lagged paths.

c Model 1c: constrained autoregressive paths.

d Model 1d: constrained all paths.

### The Longitudinal Mediating Role of Sleep Disturbances

**Figure 3.**
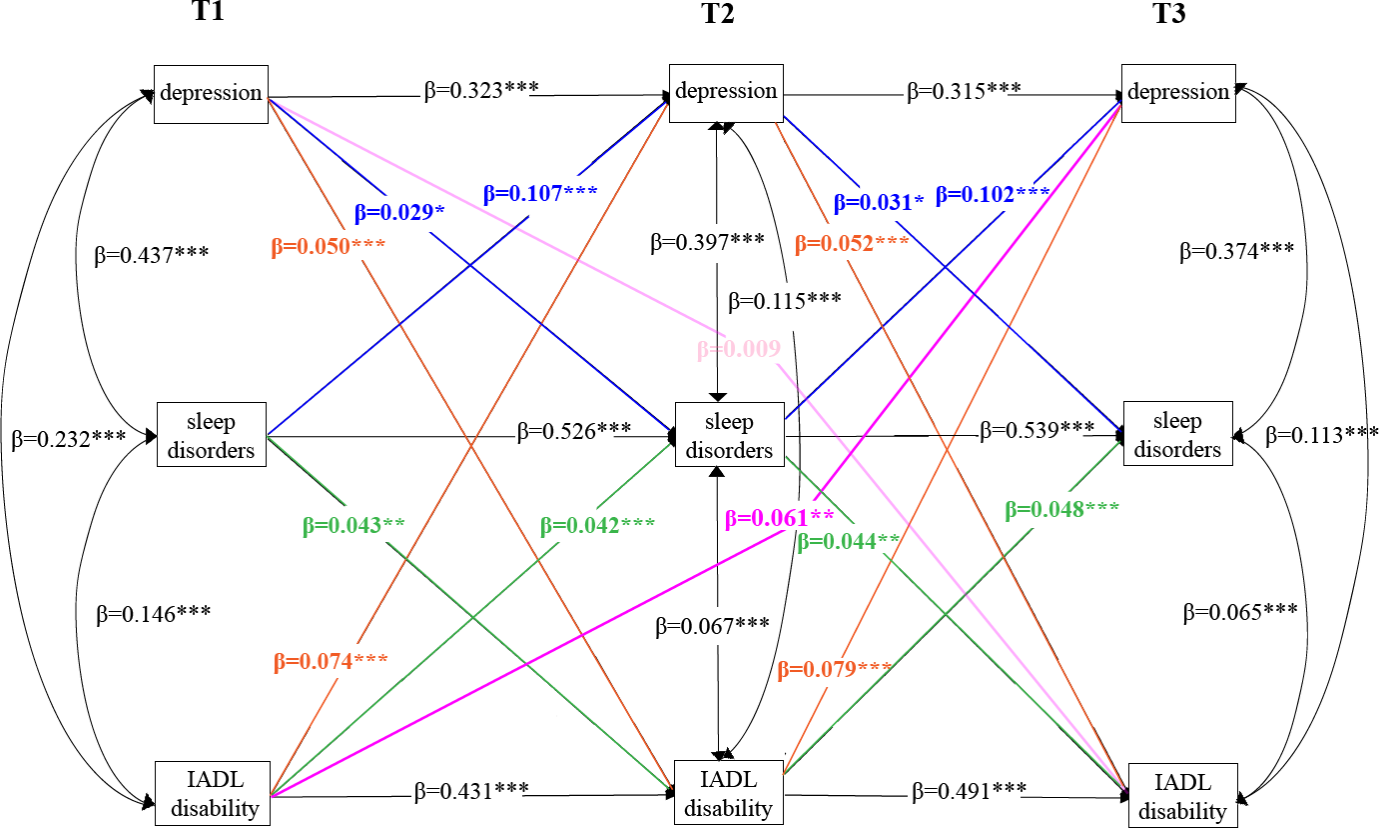
Longitudinal mediating effect of sleep disturbances in the bidirectional relationship between depressive symptoms and disability in instrumental activities of daily living (IADLs). T1: 2015; T2: 2018; T3: 2020. *P<.05, **P<.01, ***P<.001.

As Figure 3 shows, after adding 2 indirect paths that shared sleep disturbances as a potential mediator and adjustment for control variables, Model 2d still fitted the data adequately (RMSEA=0.050, SRMR=0.028, CFI=0.921, TLI=0.909). The fit indices for all 4 models are summarized in Multimedia Appendix 4. Similarly, we found cross-lagged effects of significant paths from depressive symptoms to disability in IADLs at T2 (*β*=0.050, *P*<.001) and T3 (*β*=0.052, *P*<.001) and from disability in IADLs to depressive symptoms at T2 (*β*=0.074, *P*<.001) and T3 (*β*=0.079, *P*<.001), which slightly reduced in size compared with those in Model 1d (Table 3). The cross-lagged effect of disability in IADLs on depression was still greater than the impact of depression on disability in IADLs.

Our analysis identified 2 indirect pathways from sleep disturbances. First, higher levels of prior depressive symptoms were found to be a greater risk of more severe sleep disturbances at T2 (*β*=0.029, *P*=.02) and T3 (*β*=0.031, *P*=.02), and following that, those with worse prior sleep disturbances were more likely to report higher amounts of subsequent disability in IADLs at T2 (*β*=.0043, *P*<.001) and T3 (*β*=0.044, *P*<.001). Second, greater prior disability in IADLs was a significant predictor of more severe subsequent sleep disturbances at T2 (*β*=0.042, *P*<.001) and T3 (*β*=0.048, *P*<.001); subsequently, severe prior sleep disturbances predicted greater depressive symptoms at T2 (*β*=0.107, *P*<.001) and T3 (*β*=0.102, *P*<.001).

β, standardized coefficient; CI: confidence interval.

To further test the longitudinal mediation, we computed the MacKinnon formula for calculating the mediated percentage, which is the indirect effect divided by the total effect (Multimedia Appendix 5). The indirect effects of prior disability in IADLs on depressive symptoms via sleep disturbances (*β*=0.062, SE=0.010, *P*<.001) and prior depressive symptoms on disability in IADLs via sleep disturbances (*β*=0.043, SE=0.011, *P*<.001) were significant across the 2 time intervals. Importantly, we observed that the direct effect of disability in IADLs on depression was significant (*β*=0.061, SE=0.018, *P*=.001) after considering the longitudinal mediating role of sleep disturbances, and the mediating effect of sleep disturbances reached 50.41%. Conversely, the path from depression to disability in IADLs was insignificant (*β*=0.009, SE=0.018, *P*=.61). It is worth noting that sleep disturbance was the fully mediating factor in the path from depression symptoms to disability in IADLs.

**Table 3.** Statistical results of the longitudinal mediating of sleep disturbances in the reciprocal pathway between disability in instrumental activities in daily living (IADLs) and depressive symptoms.

Variable	Autoregression estimates						Cross-lagged estimates					
	2015→2018			2018→2020			2015→2018			2018→2020		
	β (95%CI)		*P* value	β (95%CI)		*P* value	β (95%CI)		*P* value	β (95%CI)		*P* value
Disability in IADLs→sleep disturbances→depressive symptoms												
Model 2a^a^												
Disability in IADLs→sleep disturbances	0.472 (0.442-0.503)		<.001	0.466 (0.435-0.496)		<.001	0.057 (0.024-0.090)		.001	0.036 (0.004-0.068)		.03
Sleep disturbances→depressive symptoms	0.506 (0.474-0.538)		<.001	0.557 (0.526-0.588)		<.001	0.097 (0.058-0.135)		<.001	0.111 (0.073-0.150)		<.001
Disability in IADLs→depressive symptoms	0.309 (0.271-0.347)		<.001	0.327 (0.290-0.365)		<.001	0.092 (0.056-0.127)		<.001	0.067 (0.028-0.106)		.001
Model 2b^b^												
Disability in IADLs→sleep disturbances	0.470 (0.440-0.499)		<.001	0.468 (0.438-0.498)		<.001	0.042 (0.021-0.063)		<.001	0.049 (0.024-0.073)		<.001
Sleep disturbances→depressive symptoms	0.520 (0.492-0.549)		<.001	0.543 (0.514-0.572)		<.001	0.108 (0.080-0.136)		<.001	0.100 (0.074-0.127)		<.001
Disability in IADLs→depressive symptoms	0.300 (0.266-0.335)		<.001	0.335 (0.302-0.369)		<.001	0.077 (0.051-0.103)		<.001	0.082 (0.054-0.109)		<.001
Model 2c^c^												
Disability in IADLs→sleep disturbances	0.323 (0.295-0.351)		<.001	0.491 (0.466-0.517)		<.001	0.051 (0.019-0.083)		.002	0.040 (0.009-0.071)		.01
Sleep disturbances→depressive symptoms	0.523 (0.499-0.547)		<.001	0.545 (0.517-0.573)		<.001	0.100 (0.064-0.135)		<.001	0.111 (0.075-0.147)		<.001
Disability in IADLs→depressive symptoms	0.431 (0.408-0.454)		<.001	0.314 (0.285-0.343)		<.001	0.081 (0.046-0.117)		<.001	0.073 (0.035-0.112)		<.001
Model 2d^d^												
Disability in IADLs→sleep disturbances	0.431 (0.408-0.454)		<.001	0.491 (0.465-0.516)		<.001	0.042 (0.021-0.063)		<.001	0.048 (0.024-0.072)		<.001
Sleep disturbances→depressive symptoms	0.526 (0.502-0.550)		<.001	0.539 (0.512-0.566)		<.001	0.107 (0.079-0.135)		<.001	0.102 (0.075-0.129)		<.001
Disability in IADLs→depressive symptoms	0.323 (0.295-0.351)		<.001	0.315 (0.286-0.344)		<.001	0.074 (0.049-0.100)		<.001	0.079 (0.052-0.106)		<.001
Depressive symptoms→sleep disturbances→disability in IADLs												
Model 2a^a^												
Depressive symptoms→sleep disturbances	0.309 (0.271-0.347)		<.001	0.327 (0.290-0.365)		<.001	0.052 (0.016-0.088)		.005	0.048 (0.012-0.076)		.03
Sleep disturbances→disability in IADLs	0.506 (0.474-0.538)		<.001	0.557 (0.526-0.588)		<.001	0.036 (0.005-0.073)		.04	0.050 (0.012-0.088)		.009
Depressive symptoms→Disability in IADLs	0.472 (0.442-0.503)		<.001	0.466 (0.435-0.496)		<.001	0.046 (0.009-0.083)		.02	0.051 (0.012-0.090)		.01
Model 2b^b^												
Depressive symptoms→sleep disturbances	0.300 (0.266-0.335)		<.001	0.335 (0.302-0.369)		<.001	0.029 (0.004-0.054)		.02	0.030 (0.005-0.056)		.02
Sleep disturbances→disability in IADLs	0.520 (0.492-0.549)		<.001	0.543 (0.514-0.572)		<.001	0.042 (0.017-0.068)		.001	0.044 (0.017-0.071)		.001
Depressive symptoms→disability in IADLs	0.470 (0.440-0.499)		<.001	0.468 (0.438-0.498)		<.001	0.046 (0.020-0.072)		<.001	0.050 (0.022-0.078)		<.001
Model 2c^c^												
Depressive symptoms→sleep disturbances	0.323 (0.295-0.351)		<.001	0.314 (0.285-0.343)		<.001	0.049 (0.016-0.081)		.004	0.035 (0.008-0.070)		.03
Sleep disturbances→disability in IADLs	0.523 (0.499-0.547)		<.001	0.545 (0.517-0.573)		<.001	0.041 (0.004-0.078)		.03	0.047 (0.010-0.084)		.01
Depressive symptoms→disability in IADLs	0.431 (0.408-0.454)		<.001	0.491 (0.466-0.517)		<.001	0.058 (0.021-0.095)		.002	0.043 (0.005-0.082)		.03
Model 2d^d^												
Depressive symptoms→sleep disturbances	0.323 (0.295-0.351)		<.001	0.315 (0.286-0.344)		<.001	0.029 (0.004-0.054)		.02	0.031 (0.005-0.057)		.02
Sleep disturbances→disability in IADLs	0.526 (0.502-0.550)		<.001	0.539 (0.512-0.566)		<.001	0.043 (0.017-0.069)		.001	0.044 (0.018-0.071)		.001
Depressive symptoms→disability in IADLs	0.431 (0.408-0.454)		<.001	0.491 (0.465-0.516)		<.001	0.050 (0.023-0.076)		<.001	0.052 (0.024-0.080)		<.001

a Model 2a: unconstrained model.

b Model 2b: constrained cross-lagged paths.

c Model 2c: constrained autoregressive paths.

d Model 2d: constrained all paths.

### Stratified Analysis

The findings regarding the bidirectional relationship between depressive symptoms and disability in IADLs by gender, district, and empty nester status are shown in Multimedia Appendix 6. After controlling for covariates, there was no significant difference in the bidirectional relationship between depressive symptoms and disability in IADLs in empty nesters and non-empty nesters. Furthermore, in the 2 time intervals, the cross-lagged effect of higher prior depressive symptoms on adverse subsequent disability in IADLs was greater for women than for men; however, the pathway from severe disability in IADLs to greater subsequent depressive symptoms was not different for men and women. Another finding was that the effect of prior depressive symptoms was found to predict greater subsequent disability in IADLs in urban areas than in village areas, whereas the effect of prior disability in IADLs on subsequent depressive symptoms was not significant in different districts.

In general, sleep disturbance was a longitudinal mediator in the bidirectional association between depression and disability in IADLs in different empty-nester statuses, genders, and districts (MultimediaAppendices 7  8). We did not discover significant differences in the path from depressive symptoms to sleep disturbances at T2 and T3 across different empty nester statuses (*P*=.78). Nevertheless, we observed a significant difference in the effect of prior sleep disturbances on subsequent disability in IADLs (*P*=.04), which was higher in non-empty nesters (Multimedia Appendix 7). Similarly, a clear difference existed in the effect of prior depressive symptoms on subsequent sleep disturbances by gender: Women had higher cross-lagged effects (*P*=.008). In addition, we did not observe a significant difference in the path from sleep disturbances to disability in IADLs by gender (*P*=.93). Likewise, no differences were observed in the cross-lagged effects between depressive symptoms and sleep disturbances nor between sleep disturbances and disability in IADLs by district. Additionally, we found no differences in the longitudinal mediating effect of sleep disturbances in the reciprocal relationship between depressive symptoms and disability in IADLs across various empty-nester statuses, genders, and districts (Multimedia Appendix 8).

The mediating effect of sleep disturbances in the pathway from disability in IADLs to depressive symptoms was significantly stronger among empty nesters, accounting for 57.48%, compared with a lower proportion of 35.65% among non-empty nesters. We found that the mediated percentage of sleep disturbances in prior disability in IADLs on subsequent depressive symptoms among women was 31.51%. In addition, the mediating effect of sleep disturbance showed little difference across districts in older adults (Multimedia Appendix 9).

### Sensitivity Analysis

Acknowledging the potential conceptual overlap between item 7 of the CESD-10 scale (“My sleep was restless”) and our core measure of sleep disturbance, a sensitivity analysis was performed to assess the robustness of the mediation effects. All CLPMs were re-estimated using a modified CESD-9 score that excluded item 7 (MultimediaAppendices 2  3). The sensitivity analysis confirmed that all key cross-lagged paths underlying the mediation pathways remained statistically significant after removing the overlapping item. Although there was conceptual overlap between the sleep item in the CESD-10 scale and the sleep disturbance mediator, it did not fundamentally alter the main findings of this study. The longitudinal mediating role of sleep disturbances in the bidirectional relationship between depression and disability in IADLs was robust.

## Discussion

### Principal Findings

In this dynamic cohort study based on longitudinal data from older Chinese adults, we examined the bidirectional relationship between depressive symptoms and disability in IADLs mediated by sleep disturbances within a 5-year period. Moreover, we found the effect of disability in IADLs on depression was greater than the impact of depression on disability in IADLs. Notably, sleep disturbances served as the primary mediator in the path from depressive symptoms to disability in IADLs, and the mediating effect of sleep disturbances accounted for 50.41% of the association between disability in IADLs and subsequent depressive symptoms in older adults.

Whereas cross-sectional studies have presented a static view of pairwise correlations between depression, sleep, and disability in IADLs, a longitudinal analysis was better able to reveal how progressions of sleep disturbances affected the depression-disability cycle over time. The observed bidirectional association between depressive symptoms and disability in IADL aligns with previous research in older populations [15,36]. There were several potential mechanisms underlying the relationship between depressive symptoms and disability in IADLs. First, the prolonged presence of certain somatic depressive symptoms, particularly fatigue and pain, may contribute to enhanced decline of physical functioning over time [36]. Older adults with depressive symptoms are more likely to experience amplified symptom burden and complications of chronic medical conditions, both of which may increase the risks for disability. Another potential explanation is that biological changes associated with depressive symptoms, such as elevated cortisol levels and insulin resistance, may increase disability risks [37]. The severity of depression was associated with disability in IADLs but not disability in ADLs, possibly due to its impact on discretionary activities [38]. IADLs, which decline before basic ADLs, are key indicators of independence loss in older adults. We observed that the effect of disability in IADLs on depression was greater than the impact of depression on disability in IADLs; however, there are currently no studies explaining this phenomenon. In our opinion, the possible reason is the decrease in physical activity, cognitive impairment, and changes in comorbidity patterns with advancing age, all of which could lead to physical disability [39,40]. These somatic reactions make it difficult for older adults to adapt to the changes in the early stages, increasing the risk of depression. Conversely, for individuals with depression or those at a high risk of developing depression, the onset of functional disability is characterized by a certain time delay [41], resulting in a smaller effect size in the early stages. This reciprocal relationship highlights the need for comprehensive management of both physical and psychological factors in older adults to mitigate the bidirectional, vicious cycle of depression and functional disability.

Our study provided new evidence on how sleep disturbances influence the reciprocal relationship between depression and disability in IADLs. As a result, the following conclusions could be drawn. Sleep disturbances served as the primary mediator in the path from depressive symptoms to disability in IADLs, indicating a critical link between mental health and physical functioning in older adults. This underscores the need for integrated interventions that target both mental and sleep health to promote overall well-being and maintain independence in the aging population. The mediating effect of sleep disturbances accounted for 50.41% of the association between disability in IADLs and subsequent depressive symptoms in older adults. The remaining variance in the relationship may be influenced by other factors, such as cognitive decline [39], social isolation [42], or chronic health conditions [43]. Sleep is crucial for maintaining overall well-being and plays a significant role in disrupting the detrimental cycle between depression and disability in IADLs. Several mechanisms may contribute to this relationship. The bidirectional associations and co-occurrence between sleep disturbances and late-life depression demonstrated both concurrent and sequential comorbidity patterns. Therefore, sleep disturbance was not only a comorbidity of depression but also a prodromal symptom, which could predict the occurrence and outcome of depression [21]. It is important to highlight the potential impact of treatment of sleep disturbances before, during, and after depression and functional disability. Timely identification of and interventions for sleep disturbances are crucial to reducing the increasing risks of depression and functional disability in older adults.

In the subgroup analysis, our findings also highlighted the important role of sleep disturbances in the context of specific subgroups. The mediating effect of sleep disturbances in the reciprocal relationship between disability in IADLs and depressive symptoms was significantly stronger among empty nesters, who might have less awareness and ability to maintain health, which means they might not have a better quality of life [44]. Additionally, the mediating effect of sleep disturbances was more pronounced in women, which may be related to hormonal differences and greater susceptibility to sleep disturbances among women [45]. In particular, it is necessary to focus on empty nesters, provide necessary financial support and basic social security, and develop effective intervention strategies to improve the quality of life of empty nesters. We concluded that managing or treating sleep disturbances could play an important role in reducing levels of future depression and disability. Our results have implications for planning and maintaining healthy sleep environments. Specifically, the findings underscore the importance of addressing sleep disturbance as a critical component in the prevention and management of depression and functional disability among older adults. Intervention strategies should focus on creating sleep-friendly living spaces, promoting good sleep hygiene, and providing education on the impact of poor sleep on mental and physical health. Furthermore, there is an urgent need to use portable wearable devices to objectively measure sleep-related indicators, thereby identifying and conducting evidence-based clinical research to further confirm that sleep can break the detrimental cycle between depression and functional disability. By improving sleep quality, we may not only enhance the overall well-being of older individuals but also potentially reduce the burden on health care systems by preventing the exacerbation of depressive symptoms and a decline in functional abilities.

### Limitations

Although some interesting findings are presented here, there were limitations to this study. First, due to the lack of objective measurement data, the self-reported nature of the data meant that results must be interpreted with caution. There may be a tendency to over- or underestimate symptoms. It is crucial to acknowledge the limitations inherent in our assessment of sleep disturbance. Although the composite score used in this study provided a measure for our large-scale epidemiological investigation, its exclusive reliance on self-reported sleep quality and duration, without validation against established instruments such as the Pittsburgh Sleep Quality Index or objective measures, meant that our findings should be interpreted as reflecting subjective sleep experiences rather than clinically diagnosed sleep disorders. Second, the impact on specific items of disability in IADLs was not explored nor was the severity of depression differentiated. Therefore, future subgroup analyses of such data would be more informative in clarifying the underlying mechanisms. Third, we were unable to include all potential confounding factors, such as stressful life events, medication use for depression, family history of psychiatric disorders, health behaviors and conditions, and retirement information, in the analysis or adjust for them [42]. Fourth, the CLPM used in our study did not distinguish within-person and between-person variance, which is considered a limitation by some researchers [35]. The method was not applicable when the goal was to examine the association of within-person variance. In addition, this study primarily focused on older adults in China; it is unclear whether study findings may be relevant to other countries or ethnic groups.

### Conclusions

This longitudinal cohort study provided evidence for the bidirectional relationship between depressive symptoms and disability in IADLs in older adults and highlighted the mediating role of sleep disturbances in this relationship. Addressing sleep disturbances and promoting sleep health may be key components of effective interventions for depression and functional disability in older adults. Overall, public health policies could benefit from incorporating initiatives that support healthy sleep habits across the lifespan, recognizing that sleep is a fundamental pillar of health and well-being.

## Supplementary material

10.2196/76643 Multimedia Appendix 1 Additional methodology information.

10.2196/76643 Multimedia Appendix 2 Statistical results of the longitudinal mediating of sleep disturbances in the reciprocal pathway between disability in instrumental activities of daily living (IADLs) and depressive symptoms using a modified Center for Epidemiologic Studies Depression (CESD) scale score (excluding Item 7).

10.2196/76643 Multimedia Appendix 3 Statistical results of the longitudinal mediating effect of sleep disturbances in the bidirectional relationship between depressive symptoms and disability in instrumental activities of daily living (IADLs) using a modified Center for Epidemiologic Studies Depression (CESD) scale score (excluding Item 7).

10.2196/76643 Multimedia Appendix 4 Model fit indices and comparison of 4 models.

10.2196/76643 Multimedia Appendix 5 Statistical results of the longitudinal mediating effect of sleep disturbances in the bidirectional relationship between depressive symptoms and disability in instrumental activities of daily living (IADLs).

10.2196/76643 Multimedia Appendix 6 Statistical results of the bidirectional relationship between depressive symptoms and disability in instrumental activities of daily living (IADLs) by empty nester status, gender, and district.

10.2196/76643 Multimedia Appendix 7 Statistical results of the longitudinal mediating effect of sleep disturbances in the pathway from depressive symptoms to disability in instrumental activities of daily living (IADLs) by empty nester status, gender, and district.

10.2196/76643 Multimedia Appendix 8 Statistical results of the longitudinal mediating effect of sleep disturbances in the pathway from disability in instrumental activities of daily living (IADLs) to depressive symptoms by empty nester status, gender, and district.

10.2196/76643 Multimedia Appendix 9 Statistical results of the longitudinal mediating effect of sleep disturbances in the bidirectional relationship between depressive symptoms and disability in instrumental activities of daily living (IADLs) by gender, empty nester status, and district.

10.2196/76643 Checklist 1 RECORD statement checklist of items that should be reported in observational studies using routinely collected health data.

## Abbreviations

ADL: activity of daily living
CESD: Center for Epidemiologic Studies Depression
CFI: comparative fit index
CHARLS: China Health and Retirement Longitudinal Study
CLPM: cross-lagged panel model
IADL: instrumental activity of daily living
RECORD: Reporting of Studies Conducted Using Observational Routinely-Collected Health Data
RMSEA: root mean square error of approximation
SRMR: standardized root mean square residual
T1: 2015
T2: 2018
T3: 2020
TLI: Tucker-Lewis index
