# Supplementary material for: Sleep Disturbance as a Catalyst in the Cyclical Link Between Depressive Symptoms and Disability in Instrumental Activities of Daily Living in Older Chinese Adults: Longitudinal Cohort Study
Source: JMIR Aging. 2025 Nov 6;8:e76643. doi: 10.2196/76643 (PMC12591558; doi:10.2196/76643)
Supplement: Multimedia Appendix 1 [file aging-v8-e76643-s001.docx]

*Data collection and study participants*

Data were from the China Health and Retirement Longitudinal Survey (CHARLS), a nationally representative longitudinal survey of adults aged ≥ 45 years conducted in 150 counties across 28 provinces in China. Briefly, using a multistage stratified probability- proportional-to-size (PPS) sampling method to conduct a face-to-face computer-assisted personal interview (CAPI) followed every 2 years among 17,708 participants were recruited at baseline in 2011(wave 1). The inaugural wave was conducted in 2011 (wave 1), with four additional waves occurring in 2013 (wave 2), 2015 (wave 3), 2018 (wave 4), and 2020 (wave 5). It conducted follow-up visits every two years to collect data on sociodemographic and lifestyle factors and health-related information. Throughout the survey design to data collection phases, CHARLS implemented strict quality control measures, resulting in high-quality data.

The study was based on three waves of longitudinal data from the CHARLS in 2015 (T1), 2018 (T2), and 2020 (T3). Based on the study’s purpose, the exclusion criteria were as follows: (1) age<60 years old and missing data on age in 2015; (2) missing values for measures of sleep parameters, depressive symptoms and IADLs disability in 2015; (3) participants with cognitive impairment (score<1.5 standard deviation in 2015; (4) abnormal nighttime sleep duration in 2015. After the above exclusion criteria, 5658 individuals met the requirements in 2015, following the same screening process for 2018 and 2020. The database contained three rounds of complete responses from participants on demographic backgrounds, lifestyle information, sleep parameters, depressive symptoms, and IADLs disability.

*Key variables information*

*Assessment of depressive symptoms*

Depressive symptoms were measured using the 10-item centre for Epidemiologic Studies Depression Scale (CESD-10) at each wave, with 10 questions rated on a 4-point scale assessing the participant status one week prior to the interview. As a brief screening tool for depressive symptoms, the CESD-10 had shown good validity and reliability among older Chinese adults (Cronbach’s alpha=0.81) [1]. This tool consisted of 10 items assessing the frequency of mood and behavioral symptoms during the past week: (1) bothered by little things, (2) had trouble concentrating, (3) felt depressed, (4) everything was an effort, (5) felt hopeful, (6) felt fearful, (7) sleep was restless, (8) felt happy, (9) felt lonely, and (10) could not get going [2]. The 4-point scale included none of the time or rarely (<1 days), some or a little of the time (1-2 days), occasionally or a moderate amount of the time (3-4 days), and most or all of the time (5-7 days). The summed CESD-10 scores ranged from 0 to 30 (two positive worded items were reverse coded), with higher scores suggesting higher degrees of depressive symptoms.

*Assessment of IADLs disability*

For IADLs, participants were asked whether they had any difficulties in the following six activities (doing housework, preparing a hot meal, shopping, handling finances, making calls, and taking medications) (Cronbach’s alpha = 0.86) [3]. Answers were categorized into 4 types: (1) No, I don’t have any difficulty; (2) I have difficulty but still do it; (3) Yes, I have difficulty and need help; and (4) I can’t do it. All the measures were self-reported. Participants rated the extent to which they needed help for each task on a four-point scale ranging from 0= none to 3 = most of the time.

*Assessment of sleep disturbances*

Participants were assessed for sleep disturbances, specifically focusing on self-reported sleep quality and nighttime duration. Night-time sleep duration was generated by the following questions, “During the past month, how many hours of actual sleep did you get at night (average hours for one night)”? According to the classification on sleep duration from National Sleep Foundation [4], the responses were divided into four groups according to nighttime sleep duration, with scores ranging from 0 to 3. A score of 3 was assigned to participants who reported sleeping less than 5 hours or more than 10 hours per night, a score of 2 was assigned to those who reported sleeping between 5 and 6 hours or between 9 and 10 hours per night, a score of 1 was assigned to those who reported sleeping between 6 and 7 hours or between 8 and 9 hours per night, and a score of 0 was assigned to those who reported sleeping between 7 and 8 hours per night [5]. Participants were asked to rate their sleep quality using the single-item sleep quality scale. The single item in the scale was “My sleep was restless”, which had a 4-point response scale: (1) rarely or none of the time; (2) some or a little of the time (1-2 days); (3) occasionally or a moderate amount of the time (3-4 days); (4) most or all of the time (5-7 days). Participants assessed their sleep quality on a 4-point scale, from 0 rarely or none of the time) to 3 (most or all of the time).

***Covariates***

Age was a continuous variable. Gender was grouped into male and female. Marital status was divided into two groups (married or not in the marriage). Education was classified as illiteracy, primary education or less, junior high school, and high school or more. Empty nesters status was dichotomized into empty nesters or non-empty nesters. District was categorized into living in village or urban areas. Working status was categorized as government/institutions/firm, self-employed individual, farmer, and others.

Smoking status was categorized as yes or no. Drinking status was categorized as never, drinking but less than once a month, and drinking more than once a month. Cognitive function assessment tools, derived from an adapted Chinese version of the MMSE [6], included four items: orientation, memory, attention and computation, and language. The global cognitive score (range from 0 to 31) was the sum of four scores. The higher the score, the better the function.

Non-communicable diseases were obtained by asking participants if they had been diagnosed by a physician with the following fourteen types of chronic diseases: hypertension, diabetes/high blood sugar, stroke, cancer/malignant tumor, arthritis/ rheumatism, dyslipidaemia, heart problems, liver disease, kidney disease, chronic lung diseases, asthma, stomach or other digestive diseases, emotional/ nervous/ psychiatric problems, and memory-related disease. The total number of noncommunicable was calculated.

***Statistical analysis***

For the CLPM, Ulrich Orth [7] suggested that researchers should not routinely use cross-wave equality constraints on residual variances and residual covariances, but should use cross-wave equality constraints on structural coefficients (e.g., autoregressive and cross-lagged effects). Therefore, four models were constructed. The first model (Model 1a-2a) was an unconstrained. CLPM adjusted for covariates; the second model (Model 1b-2b) was a CLPM of the cross-lagged parameters was constrained; the third model (Model 1c-2c) was a CLPM of the autoregressive parameters was constrained; the fourth model (Model 1d-2d) was a CLPM of the autoregressive parameters and cross-lagged parameters were constrained.

The following indices were used to evaluate model fit for all models: the model chi-square (χ^2^), root mean square error of approximation (RMSEA), comparative fit index (CFI), standardized root mean square residual (SRMR), and Tucker-Lewis index (TLI). The RMSEA, CFI, TLI, and SRMR were prioritized due to the fact that the χ^2^ is likely to be significant in analyses with large sample sizes [8]. The recommended cut-offs for these fit indices were as follows: RMSEA < 0.08, CFI> 0.90, TLI> 0.90, SRMR < 0.08. CFI and TLI values greater than 0.95 were considered to indicate a good model fit, whereas SRMR and RMSEA values below 0.05 were considered to indicate a good fit.41 The comparisons of the models were mainly determined by the difference in CFI (ΔCFI), SRMR (ΔSRMR), and RMSEA (ΔRMSEA) between the base model and the constrained model [9]. When the ΔCFI, ΔSRMR, and ΔRMSEA were not greater than 0.01, the invariance model was acceptable. All mediations were tested with 5000 bootstrap iterations.

**References**

1. Williams MW, Li CY, Hay CC. Validation of the 10-item Center for Epidemiologic Studies Depression Scale Post Stroke. J Stroke Cerebrovasc Dis 2020;29:105334.
2. Tian F, Yang H, Pan J. Association between functional disability and long-term trajectories of depressive symptoms: Evidence from the China Health and Retirement Longitudinal Study. J Affect Disord 2022;310:10–16.
3. Castillo CH. The Validation of the Hong Kong Chinese Version of the Lawton Instrumental Activities of Daily Living Scale for Institutionalized Elderly Persons. OTJR: Occupation Participation and Health 2002:147.
4. Hirshkowitz M, Whiton K, Albert SM, et al. National Sleep Foundation's sleep time duration recommendations: methodology and results summary. Sleep Health 2025;1:40–43.
5. Fan S, Wang Q, Zheng F, et al. Depression as a Mediator and Social Participation as a Moderator in the Bidirectional Relationship Between Sleep Disorders and Pain: Dynamic Cohort Study. JMIR Public Health Surveill 2023;9:e48032.
6. Zhou L, Ma X, Wang W. Relationship between Cognitive Performance and Depressive Symptoms in Chinese Older Adults: The China Health and Retirement Longitudinal Study (CHARLS). J Affect Disord 2021;281:454–458.
7. Orth U, Clark DA, Donnellan MB, et al. Testing prospective effects in longitudinal research: Comparing seven competing cross-lagged models. J Pers Soc Psychol 2021;120:1013–1034.
8. Xia Y, Yang Y. RMSEA, CFI, and TLI in structural equation modeling with ordered categorical data: The story they tell depends on the estimation methods. Behav. Res. Methods 2019;51:409–428
9. Newsom JT. Structural Models for Binary Repeated Measures: Linking Modern Longitudinal Structural Equation Models to Conventional Categorical Data Analysis for Matched Pairs. Struct Equ Modeling 2017;24:626–635.
