# Supplementary material for: Sleep Disturbance as a Catalyst in the Cyclical Link Between Depressive Symptoms and Disability in Instrumental Activities of Daily Living in Older Chinese Adults: Longitudinal Cohort Study
Source: JMIR Aging. 2025 Nov 6;8:e76643. doi: 10.2196/76643 (PMC12591558; doi:10.2196/76643)
Supplement: Multimedia Appendix 2 [file aging-v8-e76643-s002.docx]

**Multimedia Appendix 2.** Statistical results of the longitudinal mediating of sleep disturbance in the pathway reciprocal pathway between IADLs disability and depressive symptoms using a modified CESD Score (excluding Item 7).

| **Variable** | **Autoregression estimates** | | | | | | **Cross-lagged estimates** | | | | | |
| --- | --- | --- | --- | --- | --- | --- | --- | --- | --- | --- | --- | --- |
|  | **2015→2018** | | | **2018→2020** | | | **2015→2018** | | | **2018→2020** | | |
|  | **β** | **95%CI** | ***p* value** | **β** | **95%CI** | ***p* value** | **β** | **95%CI** | ***p* value** | **β** | **95%CI** | ***p* value** |
| **Depressive symptoms→sleep disturbance→IADLs disability** | | | | | | | | | | | | |
| **Model 2a**^a^ |  |  |  |  |  |  |  |  |  |  |  |  |
| Depressive symptoms→sleep disturbance | 0.301 | (0.265,0.337) | <0.001 | 0.321 | (0.286,0.356) | <0.001 | 0.053 | (0.019,0.086) | 0.002 | 0.035 | (0.007,0.058) | 0.009 |
| Sleep disturbance→IADLs disability | 0.516 | (0.487,0.545) | <0.001 | 0.558 | (0.530,0.585) | <0.001 | 0.045 | (0.012,0.079) | 0.009 | 0.061 | (0.026,0.096) | 0.001 |
| Depressive symptoms→IADLs disability | 0.471 | (0.441,0.502) | <0.001 | 0.465 | (0.435,0.496) | <0.001 | 0.046 | (0.011,0.080) | 0.009 | 0.050 | (0.013,0.086) | 0.007 |
| **Model 2b**^b^ |  |  |  |  |  |  |  |  |  |  |  |  |
| Depressive symptoms→sleep disturbance | 0.318 | (0.291,0.344) | <0.001 | 0.306 | (0.278,0.333) | <0.001 | 0.031 | (0.008,0.054) | 0.007 | 0.032 | (0.009,0.056) | 0.007 |
| Sleep disturbance→IADLs disability | 0.529 | (0.507,0.551) | <0.001 | 0.549 | (0.523,0.574) | <0.001 | 0.052 | (0.028,0.076) | <0.001 | 0.055 | (0.030,0.079) | <0.001 |
| Depressive symptoms→IADLs disability | 0.430 | (0.407,0.453) | <0.001 | 0.491 | (0.465,0.516) | <0.001 | 0.046 | (0.022,0.070) | <0.001 | 0.049 | (0.023,0.075) | <0.001 |
| **Model 2c**^c^ |  |  |  |  |  |  |  |  |  |  |  |  |
| Depressive symptoms→sleep disturbance | 0.318 | (0.291,0.344) | <0.001 | 0.306 | (0.278,0.333) | <0.001 | 0.053 | (0.020,0.085) | <0.001 | 0.032 | (0.008,0.057) | 0.011 |
| Sleep disturbance→IADLs disability | 0.529 | (0.507,0.551) | <0.001 | 0.549 | (0.523,0.574) | <0.001 | 0.052 | (0.017,0.086) | 0.003 | 0.057 | (0.022,0.091) | 0.001 |
| Depressive symptoms→IADLs disability | 0.43 | (0.407,0.453) | <0.001 | 0.491 | (0.465,0.516) | <0.001 | 0.058 | (0.024,0.092) | 0.001 | 0.042 | (0.007,0.078) | 0.020 |
| **Model 2d**^d^ |  |  |  |  |  |  |  |  |  |  |  |  |
| Depressive symptoms→sleep disturbance | 0.317 | (0.291,0.344) | <0.001 | 0.306 | (0.279,0.334) | <0.001 | 0.031 | (0.008,0.054) | 0.007 | 0.033 | (0.009,0.057) | 0.007 |
| Sleep disturbance→IADLs disability | 0.531 | (0.509,0.553) | <0.001 | 0.544 | (0.519,0.569) | <0.001 | 0.053 | (0.029,0.078) | <0.001 | 0.055 | (0.030,0.079) | <0.001 |
| Depressive symptoms→IADLs disability | 0.431 | (0.408,0.454) | <0.001 | 0.490 | (0.464,0.516) | <0.001 | 0.049 | (0.025,0.074) | <0.001 | 0.051 | (0.026,0.077) | <0.001 |
| **IADLs disability→sleep disturbance→depressive symptoms** | | | | | | | | | | | | |
| **Model 2a**^a^ |  |  |  |  |  |  |  |  |  |  |  |  |
| IADLs disability→sleep disturbance | 0.471 | (0.441,0.502) | <0.001 | 0.465 | (0.435,0.496) | <0.001 | 0.056 | (0.023,0.089) | 0.001 | 0.035 | (0.003,0.067) | 0.030 |
| Sleep disturbance→depressive symptoms | 0.516 | (0.487,0.545) | <0.001 | 0.558 | (0.530,0.585) | <0.001 | 0.069 | (0.033,0.106) | <0.001 | 0.085 | (0.048,0.121) | <0.001 |
| IADLs disability→depressive symptoms | 0.301 | (0.265,0.337) | <0.001 | 0.321 | (0.286,0.356) | <0.001 | 0.090 | (0.053,0.126) | <0.001 | 0.069 | (0.029,0.110) | 0.001 |
| **Model 2b**^b^ |  |  |  |  |  |  |  |  |  |  |  |  |
| IADLs disability→sleep disturbance | 0.469 | (0.439,0.498) | <0.001 | 0.468 | (0.438,0.497) | <0.001 | 0.041 | (0.020,0.062) | <0.001 | 0.048 | (0.023,0.072) | <0.001 |
| Sleep disturbance→depressive symptoms | 0.525 | (0.498,0.553) | <0.001 | 0.548 | (0.521,0.575) | <0.001 | 0.080 | (0.053,0.107) | <0.001 | 0.074 | (0.049,0.099) | <0.001 |
| IADLs disability→depressive symptoms | 0.296 | (0.262,0.331) | <0.001 | 0.326 | (0.293,0.359) | <0.001 | 0.078 | (0.051,0.104) | <0.001 | 0.082 | (0.054,0.110) | <0.001 |
| **Model 2c**^c^ |  |  |  |  |  |  |  |  |  |  |  |  |
| IADLs disability→sleep disturbance | 0.430 | (0.407,0.453) | <0.001 | 0.491 | (0.465,0.516) | <0.001 | 0.050 | (0.017,0.082) | 0.003 | 0.039 | (0.008,0.071) | 0.014 |
| Sleep disturbance→depressive symptoms | 0.529 | (0.507,0.551) | <0.001 | 0.549 | (0.523,0.574) | <0.001 | 0.070 | (0.035,0.106) | <0.001 | 0.086 | (0.050,0.122) | <0.001 |
| IADLs disability→depressive symptoms | 0.318 | (0.291,0.344) | <0.001 | 0.306 | (0.278,0.333) | <0.001 | 0.080 | (0.044,0.116) | <0.001 | 0.076 | (0.036,0.116) | <0.001 |
| **Model 2d**^d^ |  |  |  |  |  |  |  |  |  |  |  |  |
| IADLs disability→sleep disturbance | 0.431 | (0.408,0.454) | <0.001 | 0.490 | (0.464,0.516) | <0.001 | 0.041 | (0.020,0.062) | <0.001 | 0.047 | (0.023,0.071) | <0.001 |
| Sleep disturbance→depressive symptoms | 0.531 | (0.509,0.553) | <0.001 | 0.544 | (0.519,0.569) | <0.001 | 0.080 | (0.053,0.106) | <0.001 | 0.075 | (0.050,0.101) | <0.001 |
| IADLs disability→depressive symptoms | 0.317 | (0.291,0.344) | <0.001 | 0.306 | (0.279,0.334) | <0.001 | 0.075 | (0.049,0.101) | <0.001 | 0.079 | (0.051,0.107) | <0.001 |

Abbreviations: β, standardized coefficient; CI: confidence interval.

^a^Model 2a: unconstrained model.

^b^Model 2b: constrained cross-lagged paths.

^c^Model 2c: constrained autoregressive paths.

^d^Model 2d:constrained all paths.
