# Supplementary material for: Sleep Disturbance as a Catalyst in the Cyclical Link Between Depressive Symptoms and Disability in Instrumental Activities of Daily Living in Older Chinese Adults: Longitudinal Cohort Study
Source: JMIR Aging. 2025 Nov 6;8:e76643. doi: 10.2196/76643 (PMC12591558; doi:10.2196/76643)
Supplement: Multimedia Appendix 4 [file aging-v8-e76643-s004.docx]

**Multimedia Appendix 4.** Model fit indices and comparison of four models.

| **Models** | **Model fits** | | | | | | | | **Pairs** | **Comparison** | | |
| --- | --- | --- | --- | --- | --- | --- | --- | --- | --- | --- | --- | --- |
|  | **χ^2^** | **df** | ***p* value** | **RMSEA** | **95% CI** | **CFI** | **TLI** | **SRMR** |  | **ΔRMSEA** | **ΔCFI** | **ΔSRMR** |
| Bidirectional relationship between depressive symptoms and IADL disability | | | | | | | | | | | | |
| Model 1a^a^ | 386.25 | 52 | <0.001 | 0.049 | (0.044,0.054) | 0.907 | 0.901 | 0.027 |  |  |  |  |
| Model 1b^b^ | 386.53 | 54 | <0.001 | 0.048 | (0.044,0.053) | 0.907 | 0.901 | 0.027 | M1b vs. M1a | 0.001 | 0.000 | 0.000 |
| Model 1c^c^ | 402.87 | 54 | <0.001 | 0.049 | (0.045,0.054) | 0.902 | 0.900 | 0.028 | M1c vs. M1a | 0.000 | 0.005 | 0.001 |
| **Model 1d**^d^ | 403.75 | 56 | <0.001 | 0.048 | (0.044,0.053) | 0.903 | 0.900 | 0.029 | M1d vs. M1a | 0.001 | 0.004 | 0.002 |
| The longitudinal mediating role of sleep disorders in the relationship between depressive symptoms and IADL disability | | | | | | | | | | | | |
| Model 2a^a^ | 658.43 | 79 | <0.001 | 0.052 | (0.049,0.056) | 0.923 | 0.915 | 0.027 |  |  |  |  |
| Model 2b^b^ | 664.71 | 85 | <0.001 | 0.050 | (0.047,0.054) | 0.923 | 0.915 | 0.028 | M2b vs. M2a | 0.002 | 0.000 | 0.001 |
| Model 2c^c^ | 677.34 | 82 | <0.001 | 0.052 | (0.048,0.056) | 0.921 | 0.917 | 0.028 | M2c vs. M2a | 0.000 | 0.002 | 0.001 |
| **Model 2d**^d^ | 683.16 | 88 | <0.001 | 0.050 | (0.047,0.054) | 0.921 | 0.909 | 0.028 | M2d vs. M2a | 0.002 | 0.002 | 0.001 |

Note: χ^2^,chi-square; df, degrees of freedom; CI: confidence interval; SRMR: standardized root mean squared residual; RMSEA: root mean square error of approximation; CFI: comparative fit index; TLI, Tucker-Lewis Index.

^a^Model 1a-2a: unconstrained model.

^b^Model 1b-2b: constrained cross-lagged paths.

^c^Model 1c-2c: constrained autoregressive paths.

^d^Model 1d-2d:constrained all paths.
