# Supplementary material for: Sleep Disturbance as a Catalyst in the Cyclical Link Between Depressive Symptoms and Disability in Instrumental Activities of Daily Living in Older Chinese Adults: Longitudinal Cohort Study
Source: JMIR Aging. 2025 Nov 6;8:e76643. doi: 10.2196/76643 (PMC12591558; doi:10.2196/76643)
Supplement: Multimedia Appendix 5 [file aging-v8-e76643-s005.docx]

**Multimedia Appendix 5.** Statistical results of the longitudinal mediating effect of sleep disturbance in the bidirectional relationship between depressive symptoms and IADLs disability.

| **Variable** | **Total effect** | | | **Direct effect** | | | **Indirect effect** | | | **Effect size (%)**^a^ |
| --- | --- | --- | --- | --- | --- | --- | --- | --- | --- | --- |
|  | **β** | **SE** | ***p* value** | **β** | **SE** | ***p* value** | **β** | **SE** | ***p* value** |  |
| **Model 2a**^b^ |  |  |  |  |  |  |  |  |  |  |
| T1 IADLs disability to T2 sleep disturbance to T3 depressive symptoms | 0.126 | 0.017 | <0.001 | 0.058 | 0.019 | 0.002 | 0.068 | 0.012 | <0.001 | 53.97 |
| T1 depressive symptoms to T2 sleep disturbance to T3 IADLs disability | 0.056 | 0.020 | 0.005 | 0.016 | 0.019 | 0.388 | 0.040 | 0.011 | <0.001 |  |
| **Model 2b**^c^ |  |  |  |  |  |  |  |  |  |  |
| T1 IADLs disability to T2 sleep disturbance to T3 depressive symptoms | 0.122 | 0.018 | <0.001 | 0.054 | 0.018 | 0.003 | 0.068 | 0.011 | <0.001 | 55.74 |
| T1 depressive symptoms to T2 sleep disturbance to T3 IADLs disability | 0.056 | 0.019 | 0.003 | 0.018 | 0.018 | 0.316 | 0.038 | 0.010 | <0.001 |  |
| **Model 2c**^d^ |  |  |  |  |  |  |  |  |  |  |
| T1 IADLs disability to T2 sleep disturbance to T3 depressive symptoms | 0.122 | 0.018 | <0.001 | 0.059 | 0.019 | 0.002 | 0.063 | 0.011 | <0.001 | 51.64 |
| T1 depressive symptoms to T2 sleep disturbance to T3 IADLs disability | 0.057 | 0.020 | 0.005 | 0.012 | 0.019 | 0.530 | 0.045 | 0.011 | <0.001 |  |
| **Model 2d**^e^ |  |  |  |  |  |  |  |  |  |  |
| T1 IADLs disability to T2 sleep disturbance to T3 depressive symptoms | 0.123 | 0.017 | <0.001 | 0.061 | 0.018 | 0.001 | 0.062 | 0.010 | <0.001 | 50.41 |
| T1 depressive symptoms to T2 sleep disturbance to T3 IADLs disability | 0.052 | 0.019 | 0.006 | 0.009 | 0.018 | 0.609 | 0.043 | 0.011 | <0.001 |  |

Note: β, standardized coefficient; SE: standard error.

^a^Effect size is the proportion mediated, which is calculated by dividing the indirect effect by the total effect.

^b^Model 2a: unconstrained model.

^c^Model 2b: constrained cross-lagged paths.

^d^Model 2c: constrained autoregressive paths.

^e^Model 2d:constrained all paths.
