# Supplementary material for: Sleep Disturbance as a Catalyst in the Cyclical Link Between Depressive Symptoms and Disability in Instrumental Activities of Daily Living in Older Chinese Adults: Longitudinal Cohort Study
Source: JMIR Aging. 2025 Nov 6;8:e76643. doi: 10.2196/76643 (PMC12591558; doi:10.2196/76643)
Supplement: Multimedia Appendix 6 [file aging-v8-e76643-s006.docx]

**Multimedia Appendix 6.** Statistical results of the bidirectional relationship between depressive symptoms and IADLs disability by empty nesters status, gender, and district.

| **Year and variable** | **Depressive symptoms→IADLs disability** | | | | | | | | **IADLs disability→depressive symptoms** | | | | | | | |
| --- | --- | --- | --- | --- | --- | --- | --- | --- | --- | --- | --- | --- | --- | --- | --- | --- |
|  | **Autoregression estimates** | | | **Cross-lagged estimates** | | | **Wald Test** | | **Autoregression estimates** | | | **Cross-lagged estimates** | | | **Wald Test** | |
|  | **β** | **95% CI** | ***p* value** | **β** | **95%CI** | ***p* value** | **Value** | ***p* value** | **β** | **95%CI** | ***p* value** | **β** | **95%CI** | ***p* value** | **Value** | ***p* value** |
| **Empty nesters status** | | | | | | | | | | | | | | | | |
| **Non-empty nesters** |  |  |  |  |  |  |  |  |  |  |  |  |  |  |  |  |
| 2015→2018 | 0.394 | (0.353,0.435) | <0.001 | 0.063 | (0.022,0.103) | 0.002 | **0.191** | **0.662** | 0.422 | (0.384,0.460) | <0.001 | 0.069 | (0.030,0.108) | <0.001 | **2.576** | **0.109** |
| 2018→2020 | 0.384 | (0.339,0.430) | <0.001 | 0.063 | (0.022,0.103) | 0.002 |  |  | 0.483 | (0.441,0.526) | <0.001 | 0.077 | (0.034,0.120) | <0.001 |  |  |
| **Empty nesters** |  |  |  |  |  |  |  |  |  |  |  |  |  |  |  |  |
| 2015→2018 | 0.356 | (0.325,0.388) | <0.001 | 0.076 | (0.048,0.105) | <0.001 |  |  | 0.439 | (0.410,0.467) | <0.001 | 0.110 | (0.079,0.141) | <0.001 |  |  |
| 2018→2020 | 0.351 | (0.317,0.385) | <0.001 | 0.083 | (0.052,0.114) | <0.001 |  |  | 0.499 | (0.467,0.530) | <0.001 | 0.113 | (0.082,0.145) | <0.001 |  |  |
| **Gender** | | | | | | | | | | | | | | | | |
| **Male** |  |  |  |  |  |  |  |  |  |  |  |  |  |  |  |  |
| 2015→2018 | 0.339 | (0.307,0.372) | <0.001 | 0.049 | (0.020,0.077) | 0.001 | **4.740** | **0.030** | 0.488 | (0.459,0.516) | <0.001 | 0.101 | (0.069,0.132) | <0.001 | **0.312** | **0.577** |
| 2018→2020 | 0.328 | (0.293,0.362) | <0.001 | 0.049 | (0.020,0.079) | 0.001 |  |  | 0.547 | (0.516,0.578) | <0.001 | 0.107 | (0.073,0.141) | <0.001 |  |  |
| **Female** |  |  |  |  |  |  |  |  |  |  |  |  |  |  |  |  |
| 2015→2018 | 0.409 | (0.370,0.447) | <0.001 | 0.102 | (0.064,0.140) | <0.001 |  |  | 0.364 | (0.326,0.402) | <0.001 | 0.085 | (0.048,0.123) | <0.001 |  |  |
| 2018→2020 | 0.413 | (0.368,0.457) | <0.001 | 0.112 | (0.070,0.154) | <0.001 |  |  | 0.423 | (0.380,0.465) | <0.001 | 0.091 | (0.051,0.132) | <0.001 |  |  |
| **District** | | | | | | | | | | | | | | | | |
| **Village** |  |  |  |  |  |  |  |  |  |  |  |  |  |  |  |  |
| 2015→2018 | 0.360 | (0.327,0.392) | <0.001 | 0.047 | (0.018.0.076) | 0.002 | **7.797** | **0.005** | 0.446 | (0.417,0.475) | <0.001 | 0.095 | (0.064,0.126) | <0.001 | **0.005** | **0.998** |
| 2018→2020 | 0.349 | (0.315,0.384) | <0.001 | 0.051 | (0.019,0.083) | 0.002 |  |  | 0.525 | (0.493,0.557) | <0.001 | 0.100 | (0.067,0.132) | <0.001 |  |  |
| **Urban** |  |  |  |  |  |  |  |  |  |  |  |  |  |  |  |  |
| 2015→2018 | 0.387 | (0.347,0.427) | <0.001 | 0.116 | (0.077,0.156) | 0.001 |  |  | 0.402 | (0.364,0.441) | <0.001 | 0.095 | (0.056,0.133) | <0.001 |  |  |
| 2018→2020 | 0.386 | (0.342,0.430) | <0.001 | 0.114 | (0.075,0.153) | <0.001 |  |  | 0.428 | (0.386,0.471) | <0.001 | 0.103 | (0.061,0.144) | <0.001 |  |  |

Note: β, standardized coefficient; CI: confidence interval.
