# Supplementary material for: Sleep Disturbance as a Catalyst in the Cyclical Link Between Depressive Symptoms and Disability in Instrumental Activities of Daily Living in Older Chinese Adults: Longitudinal Cohort Study
Source: JMIR Aging. 2025 Nov 6;8:e76643. doi: 10.2196/76643 (PMC12591558; doi:10.2196/76643)
Supplement: Multimedia Appendix 7 [file aging-v8-e76643-s007.docx]

**Multimedia Appendix 7.** Statistical results of the longitudinal mediating of sleep disturbance in the pathway from depressive symptoms to IADLs disability by empty nesters status, gender, and district.

| **Variable** | **Depressive symptoms→sleep disturbance→IADLs disability** | | | | | | | | | | | | | |
| --- | --- | --- | --- | --- | --- | --- | --- | --- | --- | --- | --- | --- | --- | --- |
|  | **Autoregression estimates** | | | | | | **Cross-lagged estimates** | | | | | | **Wald Test** | |
|  | **2015→2018** | | | **2018→2020** | | | **2015→2018** | | | **2018→2020** | | |  |  |
|  | **β** | **95%CI** | ***p* value** | **β** | **95%CI** | ***p* value** | **β** | **95%CI** | ***p* value** | **β** | **95%CI** | ***p* value** | **Value** | ***p* value** |
| **Empty nesters status** | | | | | | | | | | | | | | |
| Depressive symptoms→sleep disturbance | | | | | | | | | | | | | | |
| **Non-empty nesters** | 0.340 | (0.295,0.386) | <0.001 | 0.331 | (0.283,0.379) | <0.001 | 0.023 | (0.019,0.064) | 0.028 | 0.023 | (0.019,0.066) | 0.028 | **0.080** | **0.778** |
| **Empty nesters** | 0.311 | (0.276,0.346) | <0.001 | 0.304 | (0.268,0.341) | <0.001 | 0.029 | (0.003,0.060) | 0.042 | 0.031 | (0.004,0.064) | 0.042 |  |  |
| Sleep disturbance→IADLs disability | | | | | | | | | | | | | | |
| **Non-empty nesters** | 0.535 | (0.495,0.575) | <0.001 | 0.529 | (0.484,0.573) | <0.001 | 0.075 | (0.030,0.119) | 0.001 | 0.072 | (0.029,0.115) | 0.001 | **3.572** | **0.038** |
| **Empty nesters** | 0.521 | (0.492,0.551) | <0.001 | 0.546 | (0.512,0.580) | <0.001 | 0.024 | (0.008,0.056) | 0.014 | 0.025 | (0.008,0.059) | 0.014 |  |  |
| **Gender** | | | | | | | | | | | | | | |
| Depressive symptoms→sleep disturbance | | | | | | | | | | | | | | |
| **Male** | 0.300 | (0.264,0.336) | <0.001 | 0.291 | (0.254,0.328) | <0.001 | 0.022 | (0.014,0.030) | 0.019 | 0.022 | (0.015,0.031) | 0.019 | **7.025** | **0.008** |
| **Female** | 0.352 | (0.308,0.396) | <0.001 | 0.349 | (0.302,0.396) | <0.001 | 0.066 | (0.027,0.106) | 0.001 | 0.071 | (0.029,0.113) | 0.001 |  |  |
| Sleep disturbance→IADLs disability | | | | | | | | | | | | | | |
| **Male** | 0.525 | (0.494,0.556) | <0.001 | 0.536 | (0.501,0.570) | <0.001 | 0.046 | (0.014,0.078) | 0.005 | 0.046 | (0.014,0.077) | 0.005 | **0.008** | **0.930** |
| **Female** | 0.526 | (0.488,0.564) | <0.001 | 0.544 | (0.501,0.587) | <0.001 | 0.044 | (0.001,0.087) | 0.037 | 0.046 | (0.001,0.091) | 0.037 |  |  |
| **District** | | | | | | | | | | | | | | |
| Depressive symptoms→sleep disturbance | | | | | | | | | | | | | | |
| **Village** | 0.315 | (0.280,0.351) | <0.001 | 0.305 | (0.268,0.342) | <0.001 | 0.023 | (0.009,0.054) | 0.162 | 0.024 | (0.010,0.057) | 0.162 | **0.589** | **0.443** |
| **Urban** | 0.333 | (0.289,0.378) | <0.001 | 0.329 | (0.283,0.376) | <0.001 | 0.039 | (0.001,0.078) | 0.052 | 0.041 | (0.002,0.082) | 0.052 |  |  |
| Sleep disturbance→IADLs disability | | | | | | | | | | | | | |  |
| **Village** | 0.502 | (0.471,0.533) | <0.001 | 0.520 | (0.485,0.554) | <0.001 | 0.034 | (0.002,0.066) | 0.038 | 0.036 | (0.002,0.070) | 0.038 | **0.501** | **0.479** |
| **Urban** | 0.560 | (0.522,0.597) | <0.001 | 0.566 | (0.523,0.609) | <0.001 | 0.060 | (0.015,0.105) | 0.009 | 0.056 | (0.014,0.098) | 0.009 |  |  |

Note: β, standardized coefficient; CI: confidence interval.
