# Supplementary material for: Sleep Disturbance as a Catalyst in the Cyclical Link Between Depressive Symptoms and Disability in Instrumental Activities of Daily Living in Older Chinese Adults: Longitudinal Cohort Study
Source: JMIR Aging. 2025 Nov 6;8:e76643. doi: 10.2196/76643 (PMC12591558; doi:10.2196/76643)
Supplement: Multimedia Appendix 8 [file aging-v8-e76643-s008.docx]

**Multimedia Appendix 8.** Statistical results of the longitudinal mediating of sleep disturbance in the pathway from IADLs disability to depressive symptoms by empty nesters status, gender, and district.

| **Year** | **IADLs disability→sleep disturbance→depressive symptoms** | | | | | | | | | | | | | |
| --- | --- | --- | --- | --- | --- | --- | --- | --- | --- | --- | --- | --- | --- | --- |
|  | **Autoregression estimates** | | | | | | **Cross-lagged estimates** | | | | | | **Wald Test** | |
|  | **2015→2018** | | | **2018→2020** | | | **2015→2018** | | | **2018→2020** | | |  |  |
|  | **β** | **95%CI** | ***p* value** | **β** | **95%CI** | ***p* value** | **β** | **95%CI** | ***p* value** | **β** | **95%CI** | ***p* value** | **Value** | ***p* value** |
| **Empty nesters status** | | | | | | | | | | | | | | |
| IADLs disability→sleep disturbance | | | | | | | | | | | | | | |
| **Non-empty nesters** | 0.417 | (0.379,0.455) | <0.001 | 0.476 | (0.433,0.519) | <0.001 | 0.054 | (0.020,0.089) | 0.002 | 0.064 | (0.023,0.104) | 0.002 | **0.839** | **0.360** |
| **Empty nesters** | 0.438 | (0.409,0.466) | <0.001 | 0.498 | (0.466,0.530) | <0.001 | 0.034 | (0.007,0.060) | 0.014 | 0.038 | (0.008,0.068) | 0.014 |  |  |
| Sleep disturbance→depressive symptoms | | | | | | | | | | | | | | |
| **Non-empty nesters** | 0.535 | (0.495,0.575) | <0.001 | 0.529 | (0.484,0.573) | <0.001 | 0.126 | (0.080,0.172) | <0.001 | 0.118 | (0.074,0.163) | <0.001 | **0.873** | **0.350** |
| **Empty nesters** | 0.521 | (0.492,0.551) | <0.001 | 0.546 | (0.512,0.580) | <0.001 | 0.097 | (0.062,0.132) | <0.001 | 0.093 | (0.059,0.127) | <0.001 |  |  |
| **Gender** | | | | | | | | | | | | | | |
| IADLs disability→sleep disturbance | | | | | | | | | | | | | | |
| **Male** | 0.485 | (0.457,0.514) | <0.001 | 0.545 | (0.514,0.576) | <0.001 | 0.036 | (0.008,0.063) | 0.011 | 0.041 | (0.009,0.073) | 0.011 | **0.354** | **0.552** |
| **Female** | 0.361 | (0.323,0.399) | <0.001 | 0.420 | (0.377,0.463) | <0.001 | 0.049 | (0.016,0.083) | 0.004 | 0.056 | (0.018,0.094) | 0.004 |  |  |
| Sleep disturbance→depressive symptoms | | | | | | | | | | | | | | |
| **Male** | 0.525 | (0.494,0.556) | <0.001 | 0.536 | (0.501,0.570) | <0.001 | 0.091 | (0.055,0.127) | <0.001 | 0.086 | (0.052,0.121) | <0.001 | **1.290** | **0.256** |
| **Female** | 0.526 | (0.488,0.564) | <0.001 | 0.544 | (0.501,0.587) | <0.001 | 0.123 | (0.078,0.167) | <0.001 | 0.118 | (0.075,0.161) | <0.001 |  |  |
| **District** | | | | | | | | | | | | | | |
| IADLs disability→sleep disturbance | | | | | | | | | | | | | | |
| **Village** | 0.445 | (0.416,0.474) | <0.001 | 0.524 | (0.492,0.556) | <0.001 | 0.045 | (0.018,0.072) | 0.001 | 0.052 | (0.021,0.083) | 0.001 | **0.045** | **0.831** |
| **Urban** | 0.397 | (0.359,0.436) | <0.001 | 0.421 | (0.378,0.463) | <0.001 | 0.036 | (0.002,0.069) | 0.037 | 0.041 | (0.003,0.079) | 0.037 |  |  |
| Sleep disturbance→depressive symptoms | | | | | | | | | | | | | | |
| **Village** | 0.502 | (0.471,0.533) | <0.001 | 0.520 | (0.485,0.554) | <0.001 | 0.099 | (0.064,0.135) | <0.001 | 0.094 | (0.060,0.129) | <0.001 | **0.041** | **0.840** |
| **Urban** | 0.560 | (0.522,0.597) | <0.001 | 0.566 | (0.523,0.609) | <0.001 | 0.121 | (0.076,0.166) | <0.001 | 0.115 | (0.072,0.159) | <0.001 |  |  |

Note: β, standardized coefficient; CI: confidence interval.
