# Supplementary material for: Sleep Disturbance as a Catalyst in the Cyclical Link Between Depressive Symptoms and Disability in Instrumental Activities of Daily Living in Older Chinese Adults: Longitudinal Cohort Study
Source: JMIR Aging. 2025 Nov 6;8:e76643. doi: 10.2196/76643 (PMC12591558; doi:10.2196/76643)
Supplement: Multimedia Appendix 9 [file aging-v8-e76643-s009.docx]

**Multimedia Appendix 9.** Statistical results of the longitudinal mediating effect of sleep disturbance in the bidirectional relationship between depressive symptoms and IADLs disability by gender, empty nesters status, and district.

| **Variable** | **Total effect** | | | **Direct effect** | | | **Indirect effect** | | | **Effect size (%)^a^** |
| --- | --- | --- | --- | --- | --- | --- | --- | --- | --- | --- |
|  | **β** | **SE** | ***p* value** | **β** | **SE** | ***p* value** | **β** | **SE** | ***p* value** |  |
| **Empty nesters status** | | | | | | | | | | |
| **Non-empty nesters** | | | | | | | | | | |
| T1 depressive symptoms to T2 sleep disturbance to T3 IADLs disability | 0.050 | 0.022 | 0.011 | 0.026 | 0.029 | 0.370 | 0.024 | 0.009 | 0.021 |  |
| T1 IADLs disability to T2 sleep disturbance to T3 depressive symptoms | 0.115 | 0.029 | <0.001 | 0.074 | 0.029 | 0.011 | 0.041 | 0.017 | 0.016 | 35.65 |
| **Empty nesters** | | | | | | | | | | |
| T1 depressive symptoms to T2 sleep disturbance to T3 IADLs disability | 0.053 | 0.023 | 0.023 | 0.003 | 0.022 | 0.891 | 0.050 | 0.014 | <0.001 |  |
| T1 IADLs disability to T2 sleep disturbance to T3 depressive symptoms | 0.127 | 0.022 | <0.001 | 0.054 | 0.022 | 0.016 | 0.073 | 0.013 | <0.001 | 57.48 |
| **Gender** | | | | | | | | | | |
| **Male** |  |  |  |  |  |  |  |  |  |  |
| T1 depressive symptoms to T2 sleep disturbance to T3 IADLs disability | 0.039 | 0.015 | 0.031 | 0.014 | 0.022 | 0.540 | 0.025 | 0.001 | 0.036 |  |
| T1 IADLs disability to T2 sleep disturbance to T3 depressive symptoms | 0.100 | 0.024 | <0.001 | 0.025 | 0.027 | 0.349 | 0.075 | 0.015 | <0.001 | —— |
| **Female** |  |  |  |  |  |  |  |  |  |  |
| T1 depressive symptoms to T2 sleep disturbance to T3 IADLs disability | 0.079 | 0.032 | 0.015 | 0.011 | 0.031 | 0.726 | 0.068 | 0.018 | <0.001 |  |
| T1 IADLs disability to T2 sleep disturbance to T3 depressive symptoms | 0.146 | 0.028 | <0.001 | 0.100 | 0.029 | 0.001 | 0.046 | 0.016 | 0.003 | 31.51 |
| **District** | | | | | | | | | | |
| **Village** |  |  |  |  |  |  |  |  |  |  |
| T1 depressive symptoms to T2 sleep disturbance to T3 IADLs disability | 0.033 | 0.014 | 0.027 | 0.004 | 0.022 | 0.874 | 0.029 | 0.014 | 0.041 |  |
| T1 IADLs disability to T2 sleep disturbance to T3 depressive symptoms | 0.127 | 0.022 | <0.001 | 0.065 | 0.023 | 0.004 | 0.062 | 0.013 | <0.001 | 48.82 |
| **Urban** |  |  |  |  |  |  |  |  |  |  |
| T1 depressive symptoms to T2 sleep disturbance to T3 IADLs disability | 0.100 | 0.031 | 0.001 | 0.035 | 0.029 | 0.226 | 0.065 | 0.017 | <0.001 |  |
| T1 IADLs disability to T2 sleep disturbance to T3 depressive symptoms | 0.112 | 0.028 | <0.001 | 0.053 | 0.029 | 0.036 | 0.059 | 0.016 | <0.001 | 52.68 |

Note: β, standardized coefficient; SE: standard error.

^a^Effect size is the proportion mediated, which is calculated by dividing the indirect effect by the total effect.
