# Supplementary material for: Sleep Disturbance as a Catalyst in the Cyclical Link Between Depressive Symptoms and Disability in Instrumental Activities of Daily Living in Older Chinese Adults: Longitudinal Cohort Study
Source: JMIR Aging. 2025 Nov 6;8:e76643. doi: 10.2196/76643 (PMC12591558; doi:10.2196/76643)
Supplement: Checklist 1 [file aging-v8-e76643-s010.docx]

**Table S1.** RECORD statement-checklist of items that should be reported in observational studies using routinely collected health data.

|  | **Item**  **No.** | **STROBE items** | **Location in**  **manuscript where items are reported** | **RECORD items** | **Location in**  **manuscript**  **where items are reported** |
| --- | --- | --- | --- | --- | --- |
| **Title and abstract** | | | | | |
|  | 1 | (a) Indicate the study’s design with a commonly used term in the title or the abstract (b)  Provide in the abstract an  informative and balanced  summary of what was done and what was found | Title page 1  Abstract page 2 | RECORD 1.1: The type of data used should be specified in the title or  abstract. When possible, the name of the databases used should be included.  RECORD 1.2: If applicable, the geographic region and timeframe within which the study took place should be reported in the title or abstract.  RECORD 1.3: If linkage between  databases was conducted for the study, this should be clearly stated in the title or abstract. | Title page 1  Abstract page 2 |
| **Introduction** | | | | | |
| Background rationale | 2 | Explain the scientific  background and rationale for the investigation being reported | Introduction  Paragraph 1-4 |  | Introduction  Paragraph 1-4 |
| Objectives | 3 | State specific objectives, including any prespecified hypotheses | Introduction  Paragraph 5 |  | Introduction  Paragraph 5 |
| **Methods** | | | | | |
| Study Design | 4 | Present key elements of study design early in the paper | Methods |  | Methods |
| Setting | 5 | Describe the setting, locations, and relevant dates, including  periods of recruitment, exposure, follow-up, and data collection | Data collection and study participants,  Figure 1 |  | Data collection and study participants, Supplemental file 1, Figure 1 |

| Participants | 6 | *(a) Cohort study* - Give the  eligibility criteria, and the  sources and methods of selection of participants. Describe  methods of follow-up  *Case-control study* - Give the  eligibility criteria, and the  sources and methods of case  ascertainment and control  selection. Give the rationale for the choice of cases and controls *Cross-sectional study* - Give the eligibility criteria, and the  sources and methods of selection of participants  *(b) Cohort study* - For matched studies, give matching criteria and number of exposed and  unexposed  *Case-control study* - For  matched studies, give matching criteria and the number of  controls per case | Data collection and study participants,  Figure 1 | RECORD 6.1: The methods of study population selection (such as codes or algorithms used to identify subjects) should be listed in detail. If this is not possible, an explanation should be  provided.  RECORD 6.2: Any validation studies of the codes or algorithms used to  select the population should be  referenced. If validation was conducted for this study and not published  elsewhere, detailed methods and results should be provided.  RECORD 6.3: If the study involved  linkage of databases, consider use of a flow diagram or other graphical display to demonstrate the data linkage  process, including the number of individuals with linked data at each stage. | Data collection and study participants, Supplemental file 1, Figure 1 |
| --- | --- | --- | --- | --- | --- |
| Variables | 7 | Clearly define all outcomes,  exposures, predictors, potential confounders, and effect  modifiers. Give diagnostic criteria, if applicable. | Measurements and Covariates, Supplemental file 2, 3 | RECORD 7.1: A complete list of codes and algorithms used to classify  exposures, outcomes, confounders, and effect modifiers should be provided. If  these cannot be reported, an  explanation should be provided. | Measurements and Covariates, Supplemental file 2, 3 |
| Data sources/ measurement | 8 | For each variable of interest, give sources of data and details of methods of assessment  (measurement).  Describe comparability of  assessment methods if there is more than one group | Measurements, Supplemental file 2 |  |  |

| Bias | 9 | Describe any efforts to address potential sources of bias | Covariates, Stratified analysis |  |  |
| --- | --- | --- | --- | --- | --- |
| Study size | 10 | Explain how the study size was arrived at | Data collection and study participants, Supplemental file 1, Figure 1 |  |  |
| Quantitative variables | 11 | Explain how quantitative  variables were handled in the analyses. If applicable, describe which groupings were chosen, and why | Statistical analysis, Supplemental file 4 |  |  |
| Statistical methods | 12 | (a) Describe all statistical  methods, including those used to control for confounding  (b) Describe any methods used to examine subgroups and  interactions  (c) Explain how missing data were addressed  (d) *Cohort study* - If applicable, explain how loss to follow-up was addressed  *Case-control study* - If  applicable, explain how  matching of cases and controls was addressed  *Cross-sectional study* - If  applicable, describe analytical  methods taking account of sampling strategy  (e) Describe any sensitivity analyses | Statistical analysis, Supplemental file 4 |  |  |
| Data access and cleaning methods |  | .. |  | RECORD 12.1: Authors should  describe the extent to which the  investigators had access to the database population used to create the study  population. | Data collection and study participants, Supplemental file 1, Figure 1 |

|  |  |  |  | RECORD 12.2: Authors should  provide information on the data  cleaning methods used in the study. |  |
| --- | --- | --- | --- | --- | --- |
| Linkage |  | .. |  | RECORD 12.3: State whether the study included person-level,  institutional-level, or other data linkage across two or more databases. The  methods of linkage and methods of linkage quality evaluation should be provided. | NA |
| **Results** | | | | | |
| Participants | 13 | (a) Report the numbers of  individuals at each stage of the study (*e.g.*, numbers potentially eligible, examined for eligibility, confirmed eligible, included in the study, completing follow-up, and analysed)  (b) Give reasons for non- participation at each stage. (c) Consider use of a flow diagram | Data collection and study participants, Supplemental file 1, Figure 1 | RECORD 13.1: Describe in detail the selection of the persons included in the study (*i.e.,* study population selection) including filtering based on data  quality, data availability and linkage. The selection of included persons can be described in the text and/or by  means of the study flow diagram. |  |
| Descriptive data | 14 | (a) Give characteristics of study participants (*e.g.*, demographic, clinical, social) and information on exposures and potential  confounders  (b) Indicate the number of  participants with missing data  for each variable of interest  (c) *Cohort study* - summarise  follow-up time (*e.g.*, average and total amount) | Results: Descriptive statistics |  |  |
| Outcome data | 15 | *Cohort study* - Report numbers of outcome events or summary measures over time  *Case-control study* - Report numbers in each exposure | Table 1, Supplemental Table S2 |  |  |

|  |  | category, or summary measures of exposure  *Cross-sectional study* - Report numbers of outcome events or summary measures |  |  |  |
| --- | --- | --- | --- | --- | --- |
| Main results | 16 | (a) Give unadjusted estimates and, if applicable, confounder- adjusted estimates and their  precision (e.g., 95% confidence interval). Make clear which  confounders were adjusted for and why they were included  (b) Report category boundaries when continuous variables were categorized  (c) If relevant, consider  translating estimates of relative risk into absolute risk for a  meaningful time period | Results, Figure 2, 3, Supplemental Table S3, S4, S5, S6 |  |  |
| Other analyses | 17 | Report other analyses done— e.g., analyses of subgroups and interactions, and sensitivity  analyses | Results: Stratified analysis, Supplemental Table S7, S8, S9, S10 |  |  |
| **Discussion** | | | | | |
| Key results | 18 | Summarise key results with reference to study objectives | Discussion paragraph 1 |  |  |
| Limitations | 19 | Discuss limitations of the study, taking into account sources of potential bias or imprecision.  Discuss both direction and  magnitude of any potential bias | Discussion paragraph 5 | RECORD 19.1: Discuss the  implications of using data that were not created or collected to answer the  specific research question(s). Include discussion of misclassification bias, unmeasured confounding, missing  data, and changing eligibility over  time, as they pertain to the study being reported. | Discussion paragraph 5 |
| Interpretation | 20 | Give a cautious overall interpretation of results considering objectives, | Discussion paragraph 2-4 |  |  |

|  |  | limitations, multiplicity of analyses, results from similar studies, and other relevant  evidence |  |  |  |
| --- | --- | --- | --- | --- | --- |
| Generalisability | 21 | Discuss the generalisability (external validity) of the study results | Discussion paragraph 2-4 |  |  |
| Other Information | | | | | |
| Funding | 22 | Give the source of funding and the role of the funders for the present study and, if applicable, for the original study on which the present article is based | Funding |  |  |
| Accessibility of protocol, raw data, and  programming code |  | .. | NA | RECORD 22.1: Authors should  provide information on how to access any supplemental information such as the study protocol, raw data, or  programming code. | NA |

*Reference: Benchimol EI, Smeeth L, Guttmann A, Harron K, Moher D, Petersen I, Sørensen HT, von Elm E, Langan SM, the RECORD Working Committee. The REporting of studies Conducted using Observational Routinely-collected health Data (RECORD) Statement. *PLoS Medicine* 2015; in press.

*Checklist is protected under Creative Commons Attribution [(CC BY)](http://creativecommons.org/licenses/by/4.0/) license.
